# Effect of Social Influence on Effort-Allocation for Monetary Rewards

**DOI:** 10.1371/journal.pone.0126656

**Published:** 2015-05-11

**Authors:** Jodi M. Gilman, Michael T. Treadway, Max T. Curran, Vanessa Calderon, A. Eden Evins

**Affiliations:** 1 Center for Addiction Medicine, Massachusetts General Hospital, Boston, Massachusetts, United States of America; 2 Harvard Medical School, Department of Psychiatry, Boston, Massachusetts, United States of America; 3 Emory University, Department of Psychology, Atlanta, Georgia, United States of America; Inserm, FRANCE

## Abstract

Though decades of research have shown that people are highly influenced by peers, few studies have directly assessed how the value of social conformity is weighed against other types of costs and benefits. Using an effort-based decision-making paradigm with a novel social influence manipulation, we measured how social influence affected individuals’ decisions to allocate effort for monetary rewards during trials with either high or low probability of receiving a reward. We found that information about the effort-allocation of peers modulated participant choices, specifically during conditions of low probability of obtaining a reward. This suggests that peer influence affects effort-based choices to obtain rewards especially under conditions of risk. This study provides evidence that people value social conformity in addition to other costs and benefits when allocating effort, and suggests that neuroeconomic studies that assess trade-offs between effort and reward should consider social environment as a factor that can influence decision-making.

## Introduction

Many decisions throughout life require us to weigh the amount of effort we are willing to expend to obtain a reward, versus the probability that the reward will successfully be obtained. When making a decision to expend high versus low effort, the decision-maker must evaluate both the reward magnitude of different options and the likelihood of obtaining the reward [1]. People are generally averse to choices that are considered risky [2] or ambiguous [3], and therefore are less willing to expend effort if a reward is unlikely (e.g. [4]). However, an often-ignored variable in effort-based decision-making is the extent to which these preferences can be modified by social input. We live in a social world, and most decisions are influenced by those around us. Yet, most value-based decision-making studies are conducted on participants in isolation.

Research has consistently reported that people can exhibit marked changes in attitudes and behaviors depending on peer groups (e.g. [5]). A long line of research, called social modeling, argues that people directly adjust their behavior to that of others (e.g. [6]). Social conformity occurs when one changes their behavior to match that of others [7], and adolescents and young adults appear to be especially vulnerable to social conformity [8]. The phenomenon of social conformity was first studied by psychologist Solomon Asch in the 1950s, who showed that people would often conform to the judgments of others, even when those judgments were incorrect [9–11]. This finding has been replicated in experiments manipulating group size, fear, unanimity, ethnicity, status in group, and judgment difficulty (see [12] for review). Recently we showed that in a delay-discounting task, young adults had a higher rate of impulsive choices after exposure to impulsive peer influence [13].

Despite a large body of research on this topic, few empirical studies have been conducted to more precisely determine the nature of these changes. In this study, we tested whether the decision to expend effort, with high or low likelihood of reward, could be increased by exposure to peers who chose to expend effort in the same situation. We introduced a social manipulation to an effort-based decision-making paradigm called the Effort-Expenditure for Rewards Task (‘‘EEfRT”)[4]. In this task, participants were asked to choose whether to expend high or low levels of effort in order to obtain rewards at two levels of probabilities: a high likelihood of reward receipt (75% chance of obtaining a reward), or low likelihood of reward receipt (25% chance). In previous work using the EEfRT task, it was shown that low-probability trials in particular are susceptible to individual differences in reward processing [4], and to variance in human dopamine function [14].

The influence of peer groups on effort is a particularly important topic of research, as studies have shown that peer influence effects are relevant to adolescents’ academic motivation and achievement [15, 16]. Peer influence can contribute to both reduced [17, 18] and increased effort in schoolwork [19]. Additionally, there has been growing recognition that alterations in the salience of social cues may play a critical role across a number of psychological disorders associated with motivational deficits, including schizophrenia [20, 21], depression [22, 23] and autism spectrum disorders [24, 25].

We hypothesized that (1) participants would choose to expend less effort during trials with a low-probability of reward, and (2) participants’ decisions to expend effort, particularly during the low-probability trials, would be influenced by the choices of peers such that the number of high-effort choices could be increased when peers made high-effort choices.

## Method

### Participants

Fifty young adults (28 men, 22 women), ages 18–25 (mean age = 20.75, SD = 1.96) participated in this study. Participants were medically healthy, with no history of psychiatric disorders (verified by the Structured Clinical Interview for DSM-IV (SCID)[26]. This sample size was estimated based on those used in other human effort-based decision-making studies using similar paradigms (e.g. [4, 27]).

### Study Procedures

The consent procedure and all study procedures were approved by the Partners Human Research Committees (PHRC) (consisting of the Institutional Review Boards (IRBs) of the Brigham and Women’s / Faulkner Hospital, Massachusetts General Hospital (MGH), McLean Hospital and North Shore Medical Center (NSMC)). Participants provided written informed consent prior to initiation of study procedures. During the consent procedure, participants were told that they were participating in a study on judgment and decision-making, but were not told that the study assessed social influence. Before beginning the task, participants were given a binder of 32 color photographs (16 of each gender) taken from the Texas Center for Vital Longevity at University of Texas, Dallas (happy expressions; ages 18–29) [28] and the Max Plank FACES database (happy expressions, young adults age 19–31)[29]. The following script was read:

*We are going to show you photos of other people who have participated in this experiment*. *In some of the games you’ll play*, *you will get to see their answers*. *Please choose 8 people whose answers you would like to see*.

**Fig 1:**
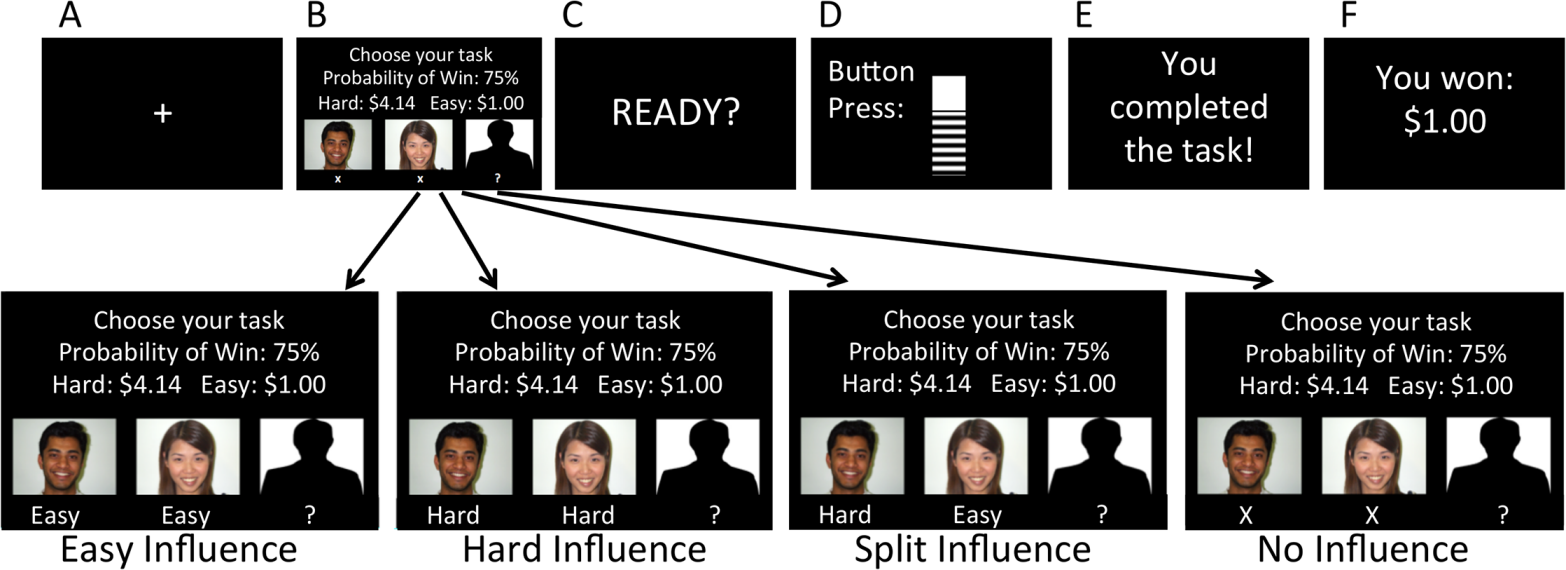
Depiction of Social Influence Manipulation to EEfRT. **Panel A** (presented for 2-seconds) showed a cross hair, indicating that a new trial was going to begin. **Panel B** (presented for 8 seconds total) was divided into several components. For three seconds, participants were shown the conditions of the trial; whether the trial had a high or low probability of receiving a reward, and the reward magnitude. Then, for two seconds, they were shown photos of peers and peer responses under each photo. There were four possible types of influence, shown from left to right in the (lower panel): Easy, Hard, Split, or None. Next, participants were then shown a bank silhouette for 2 seconds, during which they were asked to make their choice. Finally, after selecting an option, they were shown the option they chose for 1 second. **Panel C** showed a 2-second “Ready” screen, alerting the participant that the button presses were about to being. In **Panel D**, the participant completed the button press task. In **Panel E**, the participant was shown whether the task was successfully completed. In **Panel F**, the participant was shown whether or not the trial was a “win” trial. Please see Methods for detail.

The photographs chosen by participants (hereafter referred to as ‘peers’) were then presented during the task (see Fig 1). To increase believability of the paradigm, we asked the participants if we could take their photographs to be added to the binder of previous participants [13]. (Photographs of participants were not actually shown to subsequent participants; all photographs actually presented were from the databases listed above). Participants completed other tasks as part of a larger study on social influence and decision-making in a number of domains (e.g. [13]).

After completing study procedures, participants were debriefed, and were given the option of removing their photograph from the database. During the debriefing, all participants reported that they found the task believable.

### Motivation Task

The task used was a modified version of the Effort-Expenditure for Rewards Task (‘‘EEfRT”). In this task, participants are given an opportunity on each trial to choose between two task difficulty levels requiring different amounts of effort in order to obtain monetary rewards (Fig 1). For all trials, participants made repeated button presses, in which each button press raised the level of a virtual ‘‘bar” viewed on a computer screen. Participants were eligible to win money for each trial if they filled the bar to the ‘‘top” within a prescribed time period. Each trial presented the participant with a choice between a ‘hard task’ (high effort) and an ‘easy task’ (low effort). Successful completion of hard-task trials required the participant to press the button as quickly as they could (about 100 button presses, calibrated to 90% of the participant’s maximum keypress speed), using a finger on their non-dominant hand, within 21 seconds. Successful completion of easy-task trials required the participant to make 30 button presses, using a finger on the dominant hand, within 7 seconds. For each easy-task trial, participants were eligible to win $1.00 if they successfully completed the task. For hard-task trials, participants were eligible to win higher amounts that varied within a range of $3.00 –$3.50. Though the original EEfRT task included a greater manipulation of reward magnitude, we omitted this manipulation in order to reduce the number of variables; we kept the largest reward magnitude because in previous work, effort for large rewards was associated with individual differences in reward processing [4].

Participants were not guaranteed to win the reward if they successfully completed the task; some trials were ‘‘win” trials, in which the participant received the stated reward amount, while others were ‘‘no win” trials, in which the participant received no money for that trial. Trials had two levels of probability of receiving money: ‘‘high” (75% probability of winning money if the trial was completed successfully), and ‘‘low” (25% probability of winning money if the trial was completed successfully). (The original EEfRT task [4] had three probability levels; 12%, 50%, and 88%, but our modified version only had two levels in order to reduce the total number of trial types).

Trials began with a 2-second fixation cross, following a 3-second period in which participants were presented with information regarding the probability of receiving the reward and the reward magnitude of the hard task. Next, during a 2-second social influence manipulation, participants were shown photographs and ‘choices’ of two of the previously selected peers (see Fig 1). There were four types of influence: Easy (both peers chose ‘easy’), Hard (both peers chose ‘hard’), Split (one peer chose ‘easy,’ one chose ‘hard’), and, as a control condition, ‘None’ (responses of peers were not revealed to the participant). Since we were primarily interested in investigating social conformity, or how an individual decided to agree or disagree with a group, we did not analyze the ‘Split’ condition, which we included only to make the task believable (i.e. participants would get suspicious if there were no trials in which the peers were split). ‘Easy,’ ‘Hard,’ and ‘None’ were each presented in 30% of the trials shown; ‘Split’ was presented in 10% of trials.

After the participants saw the choices of these peers, they then had 2 seconds to make their own decision about whether to engage in a hard or an easy trial. They were then shown a 1-second ‘‘Ready” screen, and then started the button-pressing task. Following task completion, participants were shown a 2 second feedback screen informing them if the task was successfully completed, and a 2 second feedback screen stating whether they had won money for that trial (reward feedback). Participants were informed that they had twenty minutes to play as many trials as they could.

Participants were told that they would receive compensation for their participation, and in addition, that some win trials would be randomly selected at the end of the experiment, for which they would receive the actual amount won on those trials. In reality, all participants were paid the same amount of $7.00 at the end of the task. The EEfRT was programmed in Matlab (Matlab for Windows, Rel. 2007b. Mathworks Inc., Natick, MA) using the Psychtoolbox version 2.0.

### Questionnaires

Participants completed the Multidimensional Iowa Suggestibility Scale (MISS) [30], which assesses susceptibility to influence in five domains: consumer suggestibility (suggestibility to commercials, products), persuadability (changing one’s mind based on other peoples’ arguments), physiological suggestibility (feeling cold when someone else is shivering), physiological reactivity (feeling jumpy after watching a scary movie), and peer conformity (liking the same celebrities/fashion/music as friends) whose subscales were summed to compute a total suggestibility score.

### Statistical Analyses

We conducted repeated-measures ANOVAs using probability (high or low) and influence type (hard, easy, or none) as the independent variables, and percent of hard (high-effort) choices selected and reaction time as independent variables. We ran Mauchly's Test of Sphericity to test whether assumptions underlying the use of were met for repeated-measures ANOVAs; if they were not met, we reported corrected Greenhouse-Geiser estimates of F and p values. Where there was a significant interaction between probability and influence type, we analyzed the low- and high-probability trials separately for peer influence effects. If a significant *F* value was detected in the ANOVA, pairwise comparisons were examined using Tukey’s test for multiple comparisons. Effect sizes and 95% confidence intervals were computed using SPSS-19 (IBM) and Prism 6 software (GraphPad Software, Inc).

We also assess reaction time during the choice phase, which was defined as the amount of time participants expended choosing the ‘high-effort’ or ‘low-effort’ option after they had seen the choices of their peers. To determine whether the effect of social influence on the EEfRT was related to reaction time differences, we calculated an ‘influence score’ for each participant based on task behavior. The influence score was defined as the percentage of hard choices selected during *hard* influence minus percentage of hard choices selected during *easy* influence. These influence scores were then correlated with participants’ reaction time differences.

## Results

### Task Performance (Completion Rate)

Two out of 50 participants were excluded because their completion rates during the trials were less than 50%. After excluding these two participants, rates of completion were 98.6% for the easy (low-effort) trials (SD = 3.4), and 97.5% for the hard (high-effort) trials (SD = 4.0), demonstrating that participants were able to complete the hard task. There were no effects of probability or social influence on completion rates, and no interaction.

### Percentage of Hard (High-Effort) Trials Chosen

**Fig 2:**
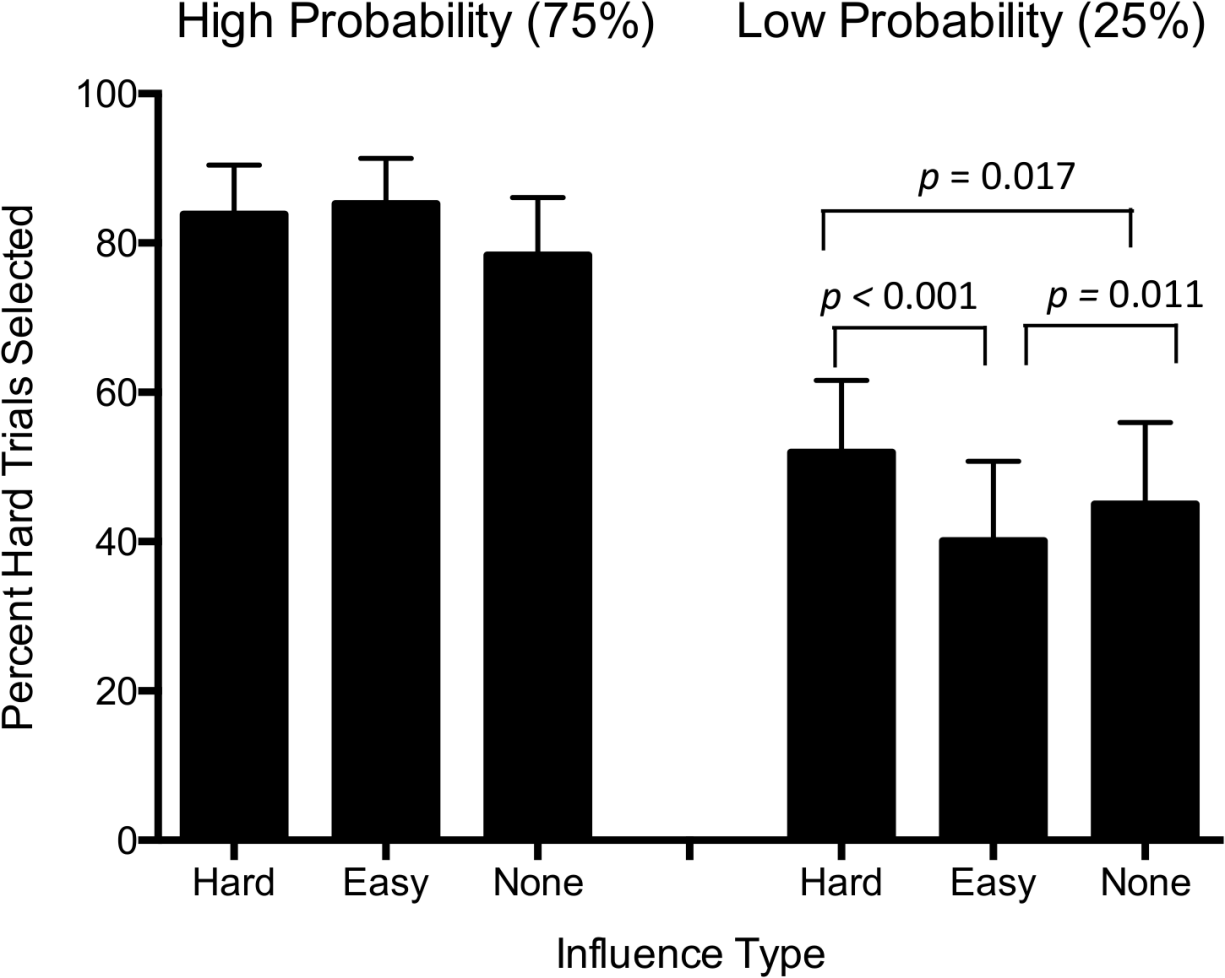
Percentage of hard (high-effort) trials selected during high (left) and low (right) probability trials during each influence type. Error bars represent 95% confidence intervals.

Means and standard deviations of choice behavior are reported in Table 1. Mauchly's Test of Sphericity indicated that the assumption of sphericity was violated, χ^2^(2) = 6.90, *p* = 0.032. We therefore are reporting Greenhouse-Geiser estimates of *F* and *p* values [31]. There was a main effect of probability on percent of hard (high-effort) choices across all influence types (*F* (1,47) = 51.9, p < 0.001, ηp^2^ = 0.53), indicating that participants selected more hard trials on the high-probability (75%) than on low-probability trials (25%). There was also a main effect of influence type across probabilities (F (2,46) = 4.69, p = 0.014, ηp^2^ = 0.17), and a significant interaction between probability and influence (F (2,46) = 7.16, p = 0.002, ηp^2^ = 0.24) (Table 2, Fig 2).

**Table 1:** Percentage of Hard Choices and Reaction Time During Influence Conditions.

	Percent Hard (High-Effort) Choices		Reaction Time (seconds)	
	*Low Probability* Mean (SD)	*High Probability* Mean (SD)	*Low Probability* Mean (SD)	*High Probability* Mean (SD)
*Hard*	53.72 (34.36)	83.0 (24.59)	0.55 (0.19)	0.50 (0.18)
*Easy*	40.21 (37.49)	83.98 (22.84)	0.46 (0.17)	0.54 (0.25)
*None*	46.18 (39.61)	77.12 (29.20)	0.51 (0.15)	0.46 (0.19)

Descriptive statistics of percent hard (high-effort) choices (left) and reaction time (right) during each of the six nested conditions.

**Table 2:** ANOVA Results of Percentage of Hard Choices and Reaction Time During Influence Conditions.

	Percent Hard (High-Effort) Choices			Reaction Time (seconds)		
	*F*	*p*	*ηp* ^*2*^	*F*	*p*	*ηp* ^*2*^
*Probability*	51.9	<0.001	0.53	NS	NS	NS
*Influence*	4.69	0.014	0.17	NS	NS	NS
*Interaction*	7.16	0.002	0.24	7.54	0.001	0.25

Results of repeated-measures ANOVAs of percent hard choices (left) and reaction time (right).

During low-probability trials (25%), peer influence had a significant effect on percent of high-effort choices made (*F* (2,46) = 9.67, *p* < 0.001, ηp^2^ = 0.30). Post-hoc tests indicated that participants chose to expend high-effort more often in trials in which their peers chose to expend high-effort than when they received low-effort peer influence (t = 4.33, *p* < 0.001). Participants also chose to expend high-effort more often in trials in which their peers chose to expend high-effort than when they received no influence (t = 2.48, *p* = 0.017), and chose fewer high-effort trials when peers chose to fewer high-effort trials compared to no influence (t = 2.65, *p* = 0.011)(Table 3; Fig 2).

**Table 3:** Pairwise Comparisons of Percentage of Hard Choices During Influence Conditions.

Influence Type	Mean Difference	SD	95% CI of difference	*t value*	*p* value
Low-Probability Trials*					
Hard vs. Easy	13.51	21.63	[7.23–19.80]	**4.33**	**<0.001**
Hard vs. None	7.54	21.09	[1.42–13.66]	**2.48**	**0.017**
Easy vs. None	-5.97	15.63	[-10.51–1.44]	**-2.65**	**0.011**
***High-Probability Trials***					
Hard vs. Easy	-0.98	19.72	[-6.71–4.75]	-0.34	0.732
Hard vs. None	5.88	25.38	[-1.49–13.25]	1.61	0.115
Easy vs. None	6.86	22.58	[3.26–0.30]	2.10	0.041

*Significance was detected across influence types using a repeated-measures ANOVA (*F* (2,46) = 9.67, *p* < 0.001, ηp^2^ = 0.30). Significant *p* values are in bold. SD, standard deviation.

During high-probability trials (75%), peer influence did not significantly affect percentage of hard choices made.

### Reaction Time

**Fig 3:**
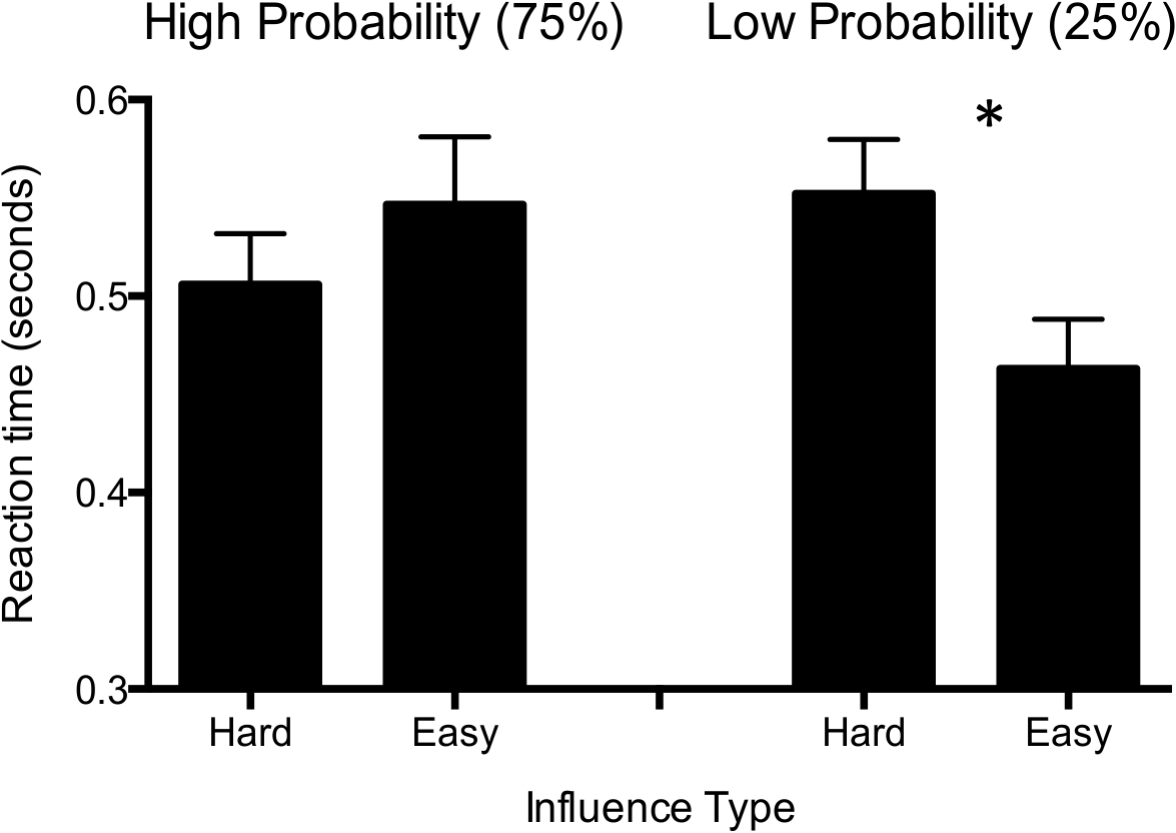
Reaction time during high (left) and low (right) probability trials during hard and easy influence. Error bars represent 95% confidence intervals. An asterisk designates a significant difference.

In addition to choice outcome, we also observed significant effects of social influence on choice reaction time (RT). Means and standard deviations are reported in Table 1. Specifically, there was a significant interaction between probability and influence (F (2, 46) = 7.54, p = 0.001, ηp^2^ = 0.25) (Table 2, Fig 3), indicating that participants had longer RTs during “unexpected” peer responses (i.e. when peers chose the easy option on high-probability trials, or chose the hard option on low-probability trials) than during “expected” peer responses. Mauchly's Test of Sphericity indicated that the assumption of sphericity was not violated for reaction time data, χ^2^(2) = 2.22, *p* = 0.33. During the low-probability trials, participants had longer RTs to the hard than the easy influence (t = 3.50, p = 0.001); this effect was not significant during high-probability trials, where there were slightly longer RTs during easy than hard influence. There were no significant main effects of probability or influence types on RT.

### Relationship between Choices and Reaction Time

**Fig 4:**
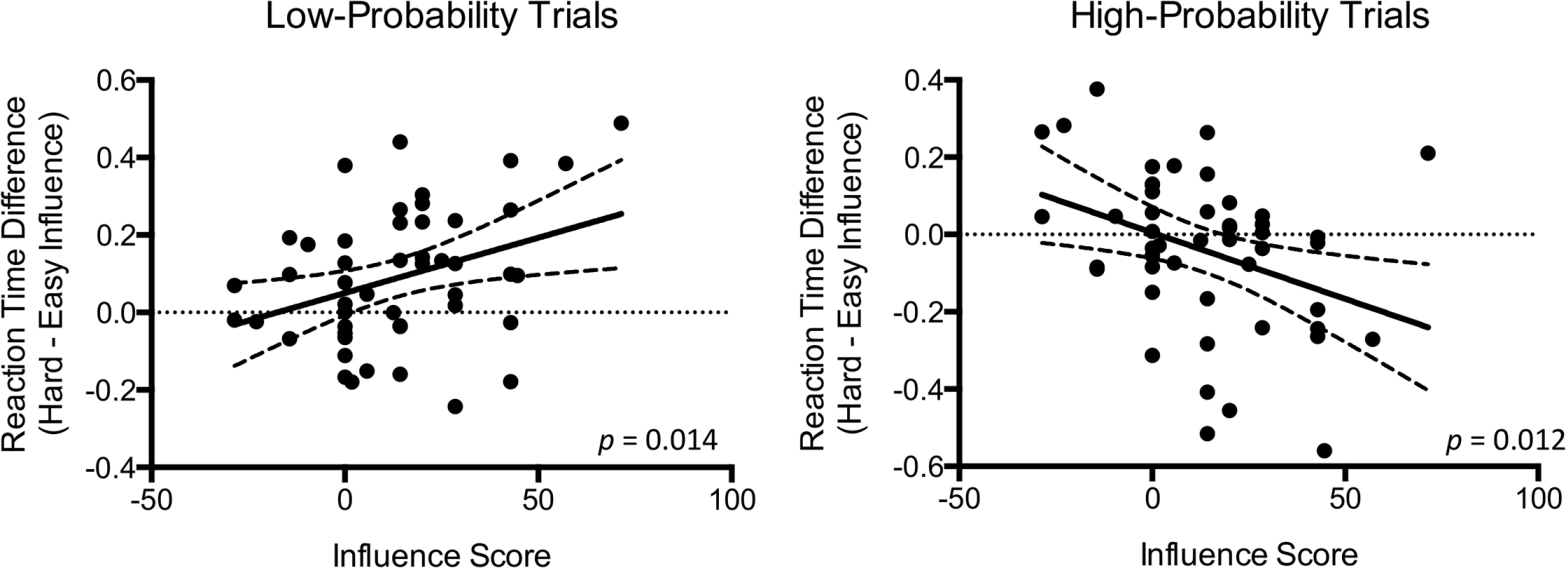
Associations between reaction time differences (reaction time during hard minus easy influence) and influence score during low- and high-probability trials. Dotted lines represent the 95% confidence interval of the associations.

We calculated an “influence score” defined as the percent of hard choices selected during hard minus easy influence during low-probability trials. This influence score correlated with differences in reaction time between hard minus easy influence during both low-probability trials (*r* = 0.35, *p* = 0.014) and high-probability trials (*r* = -0.36, *p* = 0.012) (Fig 4).

### Relationship between Task Behavior and Self-Reported Suggestibility:

A small correlation was detected between influence score and self-assessment of suggestibility on the MISS, but this was non-significant (*r* = 0.22, *p* = 0.14). Similarly, a small correlation was detected between the difference in RT for the hard versus easy low-probability trials and self-reported suggestibility, but this correlation was also non-significant (*r* = 0.23, *p* = 0.12).

## Discussion

This study demonstrates that effort-based decision-making can be modulated by social influence during conditions in which there is a low probability of obtaining a reward. Prior studies have suggested that valuation of social stimuli relies on a largely overlapping mesocorticolimbic circuitry to that of other reinforcers [32, 33]. Social conformity (or divergence) may serve as an additional value signal that is incorporated with other standard reinforcement parameters (e.g., effort, reward magnitude, probability) to derive a single subjective value for a given option [34, 35]. Consistent with this, we detected an interaction between social influence and reward probability; our social influence manipulation was generally unable to alter choices when the probability of greater reward for greater effort was high. For low probability rewards, however, we found a clear bias in favor of conformity for most participants.

Complementary effects of social influence were also observed for choice reaction times. Specifically, we found that reaction times were slowest for trials in which the social influence was “incongruent” with the expected choice given the condition probability (e.g., choosing the hard task during low probability) and fastest for “congruent” trials (e.g., choosing the hard task during high probability). Critically, these effects were related to choice outcome, such that individuals who showed a greater bias towards conformity also showed larger reaction time slowing effects during incongruent trials. Since these participants placed greater value on conformity, it is likely that incongruent trials were associated with greater conflict between probability and conformity, and required more attentional resources. Taken together, these findings help establish this task as a measure of individual differences in the value of social conformity as compared to other costs and benefits.

We found a small but non-significant correlation between task behavior and self-reported suggestibility. The absence of a stronger correlation may highlight the limitations of self-report, particularly in the domain of social influence susceptibility. Studies have found that research participants tend to under-report behaviors deemed inappropriate by researchers or other observers, and tend to over-report behaviors viewed as appropriate [36]; self-report is also affected by recall bias, and limited insight. Therefore, laboratory social manipulations may present an opportunity to more accurately measure an individual’s susceptibility to influence, and may offer insight into subtle changes in susceptibility that may not even be evident to the participant. Of note, in our larger study from which the EFfRT task was selected, influence behavior on this task correlated with influence behavior on a social influence task regarding delay discounting [13], suggesting that these behavioral laboratory tasks do measure a general construct of behavioral susceptibility to influence.

A relevant question to consider is whether it was rational for participants to expend effort for low-probability trials. Expending a great amount of effort for something with low certainty of payoff can be advantageous in specific situations, and may be a marker of ambition. Critically, peer influence only had a strong effect during low probability of reward trials, when participants may have been unsure whether it would “worth” the effort to perform the hard task. Perhaps during this uncertainty, peers influenced participants to put forth effort that they otherwise would not have.

To our knowledge, this is the first study to directly examine how opposite types of peer influence (i.e. influence to work hard vs influence to not work hard) affect peoples’ motivation to expend effort to receive rewards. It is not surprising that social influence affected participants’ choices. Social modeling argues that people adjust their behavior to that of others (e.g. [6]). Generally, peoples’ perceptions of others’ behavior have been found to be strong predictors of behavior, and this is especially true when the “others’” are thought of as a peer group [37]. Recent studies in neuroscience have implicated specific parts of the brain that are involved in decision-making during social situations [38]. In fact, some fMRI studies have indicated that going along with a group opinion engages brain structures involved with reward, such as the nucleus accumbens, that are also engaged in more traditional rewarding events, such as winning money [33, 39]. In the current study, choices that were the same as ‘peer’ choices may have been particularly rewarding to participants, which may in part explain the mechanism underlying social influence.

A limitation of this study is that social factors other than conformity, such as strategy optimization, may have affected participants’ decisions to follow group choices. In addition to the desire to fit in with a group, individuals may also go along with a majority if they believe that the group has greater knowledge than they themselves do; conforming with a group because of the desire to be ‘correct’ is termed “informational influence” [40]. Our findings cannot fully distinguish between these types of influence. This is particularly an issue in the EFfRT task, which has no optimal playing strategy; because the hard trials take longer than the easy trials to complete, there is, by design, a trade-off between based on maximizing money per unit time. It is possible that participants were agreeing with the group not only due to a conformity effect, but also to optimize strategy, and were therefore using others’ choices for information about how to get the best reward at the end of the study. Future studies could make use of non-ambiguous tasks to disentangle how different social factors contribute to decision-making.

In summary, this study utilized a novel social influence manipulation to an effort-based decision-making paradigm to demonstrate that social influence affects effort-based decision-making in young adults under conditions of low probability reward. This manipulation may be useful in objectively assessing susceptibility to peer influence, particularly in clinical populations where it may be important to distinguish between processing of social and non-social reward signals. Finally, this study demonstrates that neuroeconomic studies that assess trade-offs between effort and reward should consider social environment as a potentially important factor in the decision-making process.

## Supporting Information

S1 Dataset Raw data file. (CSV)

## References

[1] Schultz W, Preuschoff K, Camerer C, Hsu M, Fiorillo CD, Tobler PN, et al Explicit neural signals reflecting reward uncertainty. Philosophical transactions of the Royal Society of London Series B, Biological sciences. 2008;363(1511):3801–11. Epub 2008/10/03. 10.1098/rstb.2008.0152 18829433 PMC2581779

[2] Kahneman D, Tversky A Prospect theory: An analysis of decision under risk. Econometrica 1979;Econometrica 47:263–92.

[3] Camerer C, Weber M. Recent Developments in Modeling Preferences: Uncertainty and Ambiguity. Journal of Risk and Uncertainty. 1992;5:325–70.

[4] Treadway MT, Buckholtz JW, Schwartzman AN, Lambert WE, Zald DH. Worth the 'EEfRT'? The effort expenditure for rewards task as an objective measure of motivation and anhedonia. PLoS One. 2009;4(8):e6598 Epub 2009/08/13. 10.1371/journal.pone.0006598 19672310 PMC2720457

[5] Brechwald WA, Prinstein MJ. Beyond Homophily: A Decade of Advances in Understanding Peer Influence Processes. J Res Adolesc. 2011;21(1):166–79. Epub 2011/03/01. 10.1111/j.1532-7795.2010.00721.x 23730122 PMC3666937

[6] Bandura A. A Social Learning Theory. Englewood Cliffs, NJ: Prentice Hall; 1977.

[7] Cialdini RB, Goldstein NJ. Social influence: compliance and conformity. Annual review of psychology. 2004;55:591–621. Epub 2004/01/28. 10.1146/annurev.psych.55.090902.142015 . 14744228

[8] Gardner M, Steinberg L. Peer influence on risk taking, risk preference, and risky decision making in adolescence and adulthood: an experimental study. Developmental psychology. 2005;41(4):625–35. Epub 2005/08/03. 10.1037/0012-1649.41.4.625 . 16060809

[9] Asch SE. Effects of group pressure upon the modification distortion of judgments In: Guetzkow H, editor. Groups, Leadership, and Men. Pittsburgh, PA: Carnegie Press; 1951 p. 177–90.

[10] Asch SE. Social Psychology. Englewood Ciffs, NJ: Prentice Hall; 1952.

[11] Asch SE. Studies of independence and conformity: a minority of one against a unanimous majority. Psychological Monogram. 1956;70(416).

[12] Baron RS, Vandello J.A., Brunsman B. The Forgotton Variable in Conformity Research: Impact fo Task Performance on Social Influence. Journal of Personality and Social Psychology. 1996;71(5):915–27.

[13] Gilman JM, Curran MT, Calderon V, Stoeckel LE, Evins AE. Impulsive social influence increases impulsive choices on a temporal discounting task in young adults. PLoS One. 2014;9(7):e101570 10.1371/journal.pone.0101570 24988440 PMC4079280

[14] Treadway MT, Buckholtz JW, Cowan RL, Woodward ND, Li R, Ansari MS, et al Dopaminergic mechanisms of individual differences in human effort-based decision-making. The Journal of neuroscience: the official journal of the Society for Neuroscience. 2012;32(18):6170–6. Epub 2012/05/04. 10.1523/JNEUROSCI.6459-11.2012 22553023 PMC3391699

[15] Ryan AM. The peer group as a context for the development of young adolescent motivation and achievement. Child development. 2001;72(4):1135–50. Epub 2001/08/02. . 11480938 10.1111/1467-8624.00338

[16] Wentzel KR, Barry C. M., & Caldwell K. A. Friendships in middle school: Influences on motivation and school adjustment. Journal of Educational Psychology. 2004;96(195–203).

[17] Bishop JH. Why the apathy in American high schools? Educational Researcher. 1989;18:6–10.

[18] Goodlad JI. A place called school New York: McGraw-Hill; 1984.

[19] Brown BB, Clasen D. R., & Eicher S. A.. Perceptions of peer pressure, peer conformity dispositions, and self-reported be- havior among adolescents. Developmental Psychology. 1986;22:521–30.

[20] Barch DM, Dowd EC. Goal representations and motivational drive in schizophrenia: the role of prefrontal-striatal interactions. Schizophr Bull. 2010;36(5):919–34. Epub 2010/06/23. 10.1093/schbul/sbq068 20566491 PMC2930335

[21] Strauss GP, Gold JM. A new perspective on anhedonia in schizophrenia. The American journal of psychiatry. 2012;169(4):364–73. Epub 2012/03/13. 10.1176/appi.ajp.2011.11030447 22407079 PMC3732829

[22] Forbes EE, Dahl RE. Research Review: altered reward function in adolescent depression: what, when and how? Journal of child psychology and psychiatry, and allied disciplines. 2012;53(1):3–15. Epub 2011/11/29. 10.1111/j.1469-7610.2011.02477.x 22117893 PMC3232324

[23] Treadway MT, Zald DH. Parsing Anhedonia: Translational Models of Reward-Processing Deficits in Psychopathology. Curr Dir Psychol Sci. 2013;22(3):244–9. Epub 2014/04/22. 10.1177/0963721412474460 24748727 PMC3989147

[24] Dichter GS, Felder JN, Green SR, Rittenberg AM, Sasson NJ, Bodfish JW. Reward circuitry function in autism spectrum disorders. Social cognitive and affective neuroscience. 2012;7(2):160–72. Epub 2010/12/15. 10.1093/scan/nsq095 21148176 PMC3277365

[25] Chevallier C, Kohls G, Troiani V, Brodkin ES, Schultz RT. The social motivation theory of autism. Trends in cognitive sciences. 2012;16(4):231–9. Epub 2012/03/20. 10.1016/j.tics.2012.02.007 22425667 PMC3329932

[26] First MB, Spitzer, Robert L, Gibbon Miriam, and Williams, Janet B.W. Structured Clinical Interview for DSM-IV-TR Axis I Disorders, Research Version, Patient Edition. (SCID-I/P) New York: Biometrics Research, New York State Psychiatric Institute; 2002.

[27] Barch DM, Treadway MT, Schoen N. Effort, anhedonia, and function in schizophrenia: Reduced effort allocation predicts amotivation and functional impairment. Journal of abnormal psychology. 2014;123(2):387–97. Epub 2014/06/03. 10.1037/a0036299 . 24886012 PMC4048870

[28] Minear M, Park DC. A lifespan database of adult facial stimuli. Behav Res Methods Instrum Comput. 2004;36(4):630–3. Epub 2005/01/12. . 15641408 10.3758/bf03206543

[29] Troje NF, Bulthoff HH. Face recognition under varying poses: the role of texture and shape. Vision Res. 1996;36(12):1761–71. Epub 1996/06/01. . 8759445 10.1016/0042-6989(95)00230-8

[30] Kotov R, Bellman, SB, Watson, DB Multidimensional Iowa Suggestibility Scale (MISS) Brief Manual. Stoneybrook Medicine website. 2004:Available: http://medicine.stonybrookmedicine.edu/system/files/MISSBriefManual.pdf. Accessed 2014 Jun 10.

[31] Greenhouse SW, & Geisser S. On methods in the analysis of profile data. Psychometrika,. 1959; 24:95–112.

[32] Jones RM, Somerville LH, Li J, Ruberry EJ, Libby V, Glover G, et al Behavioral and neural properties of social reinforcement learning. The Journal of neuroscience: the official journal of the Society for Neuroscience. 2011;31(37):13039–45. Epub 2011/09/16. 10.1523/JNEUROSCI.2972-11.2011 21917787 PMC3303166

[33] Klucharev V, Hytonen K, Rijpkema M, Smidts A, Fernandez G. Reinforcement learning signal predicts social conformity. Neuron. 2009;61(1):140–51. Epub 2009/01/17. doi: S0896-6273(08)01020-9 [pii] 10.1016/j.neuron.2008.11.027 . 19146819

[34] Kable JW, Glimcher PW. The neurobiology of decision: consensus and controversy. Neuron. 2009;63(6):733–45. Epub 2009/09/26. 10.1016/j.neuron.2009.09.003 19778504 PMC2765926

[35] Sescousse G, Li Y, Dreher, JC. A common currency for the computation of motivational values in the human striatum. Soc Cogn Affect Neurosci 2014; May 16. 10.1093/scan/nsu074 PMC4381230 24837478

[36] Donaldson SI, Grant-Vallone EJ. Understandign Self-Report Bias in Organizational Behavior Research. Journal of Business and Psychology. 2002;17(2).

[37] Polonec LD, Major AM, Atwood LE. Evaluating the believability and effectiveness of the social norms message "most students drink 0 to 4 drinks when they party". Health Commun. 2006;20(1):23–34. Epub 2006/07/04. 10.1207/s15327027hc2001_3 . 16813486

[38] Adolphs R. Cognitive neuroscience of human social behaviour. Nat Rev Neurosci. 2003;4(3):165–78. Epub 2003/03/04. 10.1038/nrn1056 . 12612630

[39] Chein J, Albert D, O'Brien L, Uckert K, Steinberg L. Peers increase adolescent risk taking by enhancing activity in the brain's reward circuitry. Dev Sci. 2011;14(2):F1–10. Epub 2011/04/19. 10.1111/j.1467-7687.2010.01035.x 21499511 PMC3075496

[40] David BA, Turner J.C. Self-categorization principles underlying majority and minority influence. Social influence: Direct and indirect processes,. 2001; 3: 293–313.

